# Risk factors of lymph node metastasis or lymphovascular invasion for superficial esophageal squamous cell carcinoma: A practical and effective predictive nomogram based on a cancer hospital data

**DOI:** 10.3389/fmed.2022.1038097

**Published:** 2022-11-17

**Authors:** Yali Tao, Shengsen Chen, Jiangping Yu, Qiwen Shen, Rongwei Ruan, Shi Wang

**Affiliations:** Department of Endoscopy, Zhejiang Cancer Hospital, Institute of Basic Medicine and Cancer (IBMC), Chinese Academy of Sciences, Hangzhou, China

**Keywords:** lymph node metastasis, lymphovascular invasion, superficial esophageal squamous cell carcinoma, predictive model, nomogram, random forest

## Abstract

**Background:**

Lymphovascular invasion (LVI) is mostly used as a preoperative predictor to establish lymph node metastasis (LNM) prediction models for superficial esophageal squamous cell carcinoma (SESCC). However, LVI still needs to be confirmed by postoperative pathology. In this study, we combined LNM and LVI as a unified outcome and named it LNM/LVI, and aimed to develop an LNM/LVI prediction model in SESCC using preoperative factors.

**Methods:**

A total of 512 patients who underwent radical resection of SESCC were retrospectively collected. Logistic regression and least absolute shrinkage and selection operator (LASSO) regression were adopted to identify the predictive factors of LNM/LVI. Integrated discrimination improvement (IDI) and net reclassification improvement (NRI) were calculated to select the potential predictive factors from the results of LASSO and logistic regression. A nomogram for predicting LNM/LVI was established by incorporating these factors. The efficacy, accuracy, and clinical utility of the nomogram were, respectively, assessed with the area under the curve (AUC), calibration curve, and decision curve analysis (DCA). Finally, the random forest (RF) algorithm was used to further evaluate the impact of these factors included in the nomogram on LNM/LVI.

**Results:**

Tumor size, tumor location, tumor invasion depth, tumor differentiation, and macroscopic type were confirmed as independent risk factors for LNM/LVI according to the results of logistic regression, LASSO regression, IDI, and NRI analyses. A nomogram including these five variables showed a good performance in LNM/LVI prediction (AUC = 0.776). The calibration curve revealed that the predictive results of this nomogram were nearly consistent with actual observations. Significant clinical utility of our nomogram was demonstrated by DCA. The RF model with the same five variables also had similar predictive efficacy with the nomogram (AUC = 0.775).

**Conclusion:**

The nomogram was adopted as a final tool for predicting LNM/LVI because its risk score system made it more user-friendly and clinically useful than the random forest model, which can help clinicians make optimal treatment decisions for patients with SESCC.

## Introduction

Esophageal cancer is one of the most common malignant tumors on a global scale, with a very high mortality rate (1). The histopathological types of esophageal cancer are mainly classified as squamous cell carcinoma (SCC) and adenocarcinoma, and this cancer in western countries is predominantly adenocarcinoma, while in Asian countries like China is dominated by squamous cell carcinoma (1, 2). Superficial esophageal squamous cell carcinoma (SESCC) is defined as esophageal cancer with tumor cell invasion confined to the intraepithelial (Tis), mucosal (T1a), or submucosal (T1b), without respect to regional lymph node metastasis (LNM) (3).

Conventionally, esophagectomy is taken as the mainstay treatment for SESCC. However, substantial postoperative morbidity and mortality have been reported to be related to this treatment (3). Recently, with the development and improvement of endoscopic resection (ER), such as endoscopic mucosal dissection (ESD) or endoscopic mucosal resection (EMR), SESCC patients without LNM are recommended to receive ER because this treatment is a minimally invasive procedure with few complications and it also has good curative effect for LNM negative patients (4, 5). Nevertheless, if patients with SESCC have LNM, esophagectomy plus lymph node dissection needs to be considered for these patients (6, 7). Therefore, the preoperative lymph node status of patients with SESCC is a critical factor in determining the treatment strategy (8), and exploring the risk factors of lymph node metastasis is of great significance for clinical decision-making. LNM is usually assessed by endoscopic ultrasonography (EUS), contrast-enhanced computed tomography (CT), and fluorodeoxyglucose positron emission tomography (FDG-PET) in prior studies (9–11), but LNM often go undetected preoperatively by these methods because of their limited accuracy (12).

Lymphovascular invasion (LVI) is a histopathological condition in which tumor cells are found in the lymphatics or blood vessels (13). It has been identified to play a crucial role in the development of LNM (14). Besides, LVI can greatly increase the risk of local malignant tumors micrometastasis (15) and lead to poor prognosis in patients with SESCC (16). However, almost all LVI is confirmed by postoperative histopathological examination, and it is extremely difficult to assess the status of LVI before surgery (17, 18), which means that using LVI to make a preoperative prediction for LNM may be infeasible. So LNM and LVI should be integrated and considered as one outcome (it can be named LNM/LVI), and usable models for predicting the presence of LNM/LVI in patients with SESCC still have not been reported.

In this study, we aimed to explore the feasible preoperative clinical variables for LNM/LVI prediction in SESCC and to stratify LNM/LVI risk according to nomogram analysis. The development of a user-friendly clinical tool to predict which patients are at high risk of LNM/LVI will facilitate the selection of the most appropriate treatment in patients with SESCC.

## Methods

### Patient selection and data collection

**Figure 1:**
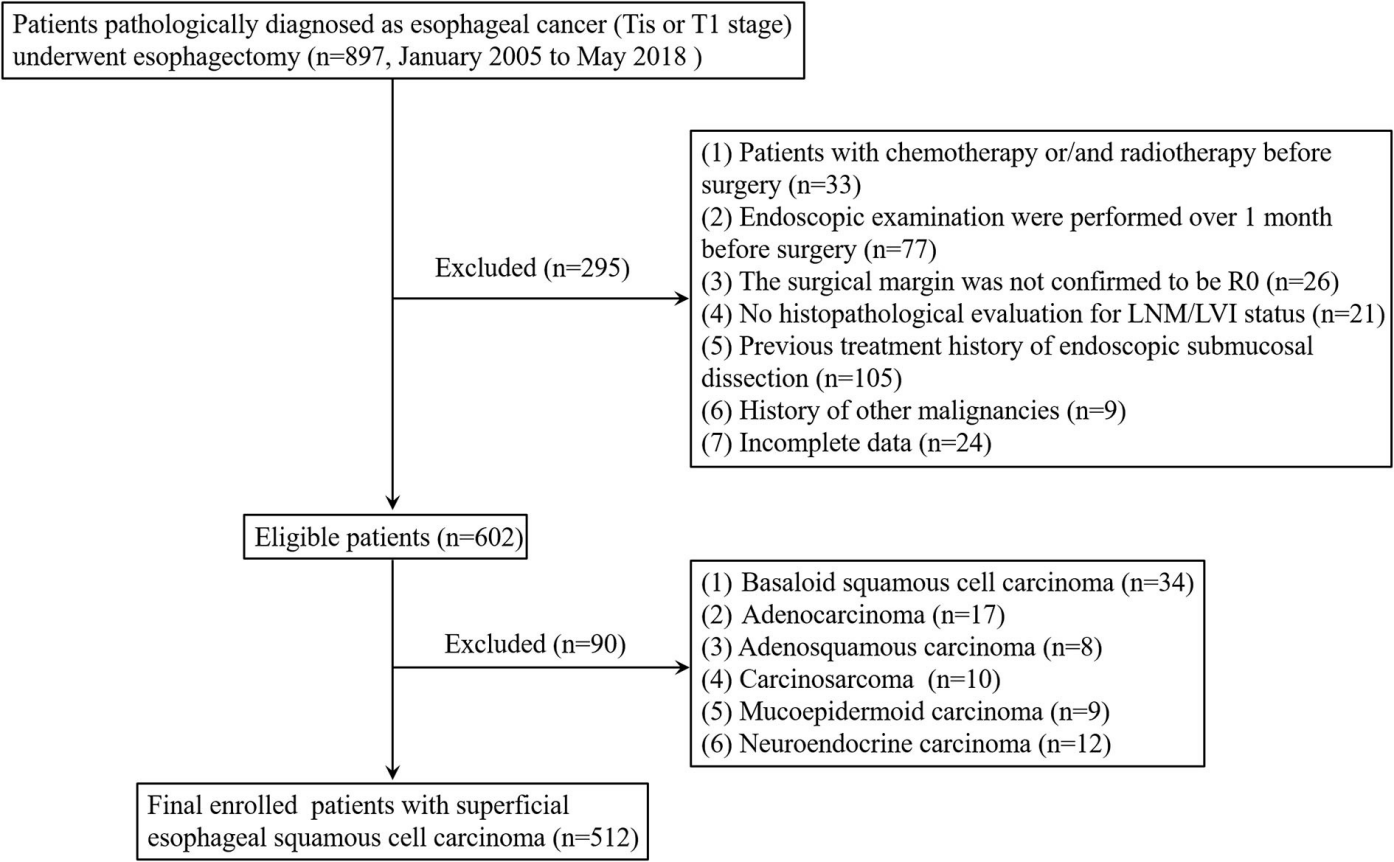
Flowchart of patients included in the analysis.

We retrospectively collected patients who were diagnosed with esophageal cancer (Tis and T1 stages) and underwent esophagectomy from January 2005 to May 2018. The exclusion criteria were as follows: (1) patients who had received radiotherapy or chemotherapy before surgery; (2) patients who had undergone endoscopy examination more than 1 month before surgery; (3) surgical margins did not reach R0; (4) LNM/LVI status had not been evaluated by histopathological examination; (5) patients already had undergone ESD before esophagectomy (patients received a supplementary surgery after ESD); (6) history of other malignant tumors; and (7) clinical data were incomplete. In addition, pathological findings of basaloid squamous cell carcinoma, adenocarcinoma, adenosquamous carcinoma, carcinosarcoma, mucoepidermoid carcinoma, and neuroendocrine carcinoma were also excluded. A total of 512 eligible patients with SESCC were finally enrolled in our study. The flowchart of patient selection is shown in Figure 1.

### Clinical variables and pathological characteristics

Basic information on admissions such as age and sex was collected. Then, circumferential extension, number of lesions, tumor location, and macroscopic morphology were extracted from the endoscopic reports. Besides, the data on tumor size, depth of invasion, LNM, and LVI were obtained from the results of postoperative histopathological examination. Here, we combined the LNM and LVI as one integrated parameter which is named LNM/LVI. LNM positive and LVI negative (LNM+/LVI–), LNM negative and LVI positive (LNM–/LVI+), and LNM positive and LVI positive (LNM+/LVI+) were considered as LNM/LVI positive. LNM/LVI negative meant LNM negative and LVI negative (LNM–/LVI–).

### Statistical analysis

Comparisons between continuous variables were performed by using the Mann–Whitney *U*-test. We compared categorical variables by using the χ^2^ test or Fisher's exact test. Variables that were significantly associated with LNM/LVI in univariate analysis or had clinical significance for LNM/LVI were incorporated into multivariate logistic regression. LASSO regression is used to reduce the dimensionality of data and screen out the most significant variables with non-zero coefficients (19). The net reclassification improvement (NRI) can be used to assess whether the classification performance of a model will improve if one important parameter is added (20). The integrated discrimination improvement (IDI) reflects the difference in the prediction probabilities of two models, that is, it can be used to evaluate the overall improvement in the predictive power of a model when a new variable is incorporated into this model (21).

A nomogram for LNM/LVI prediction was constructed by incorporating variables that were significantly correlated with an outcome event. Nomogram can proportionally convert the regression coefficients to a scale of 0–100 points (22) so that each variable in the nomogram is assigned a corresponding score, and the predicted probability can be known according to the sum of each variable point. The receiver operating characteristic (ROC) curve and the calibration curve were adopted to evaluate the predictive performance of the nomogram. Then, decision curve analysis (DCA) was performed to assess the clinical utility of this nomogram by calculating patients' net benefits at different points of threshold probabilities (23).

Additionally, the variables in the nomogram were incorporated into a random forest model to further confirm the predictive effect of these variables for LNM/LVI. The random forest algorithm is a Bagging method (ensemble learning) based on a decision tree algorithm (24). The randomly selected samples with bootstrap are used to develop decision trees, and the nodes of these trees are split based on the randomly selected best subset of explanatory variables or features (24). In a random forest, the final prediction for an event is determined according to the majority vote of prediction results generated by each tree (25). The importance of variables included in the model is generally represented by the mean decreased Gini in the output results of random forest. The greater the mean decreased Gini, the more important the variable in the random forest model (26). Finally, the ROC analysis was used to estimate the predictive ability of the random forest for LNM/LVI in patients with SESCC.

In all analyses of this study, a two-sided *p*-value of < 0.05 was considered statistically significant. All statistical analyses were performed using SPSS version 22.0 (SPSS Inc, Chicago, IL, USA) and R version 4.0.3 (The R Foundation for Statistical Computing, Vienna, Austria).

## Results

### Clinicopathological characteristics

**Figure 2:**
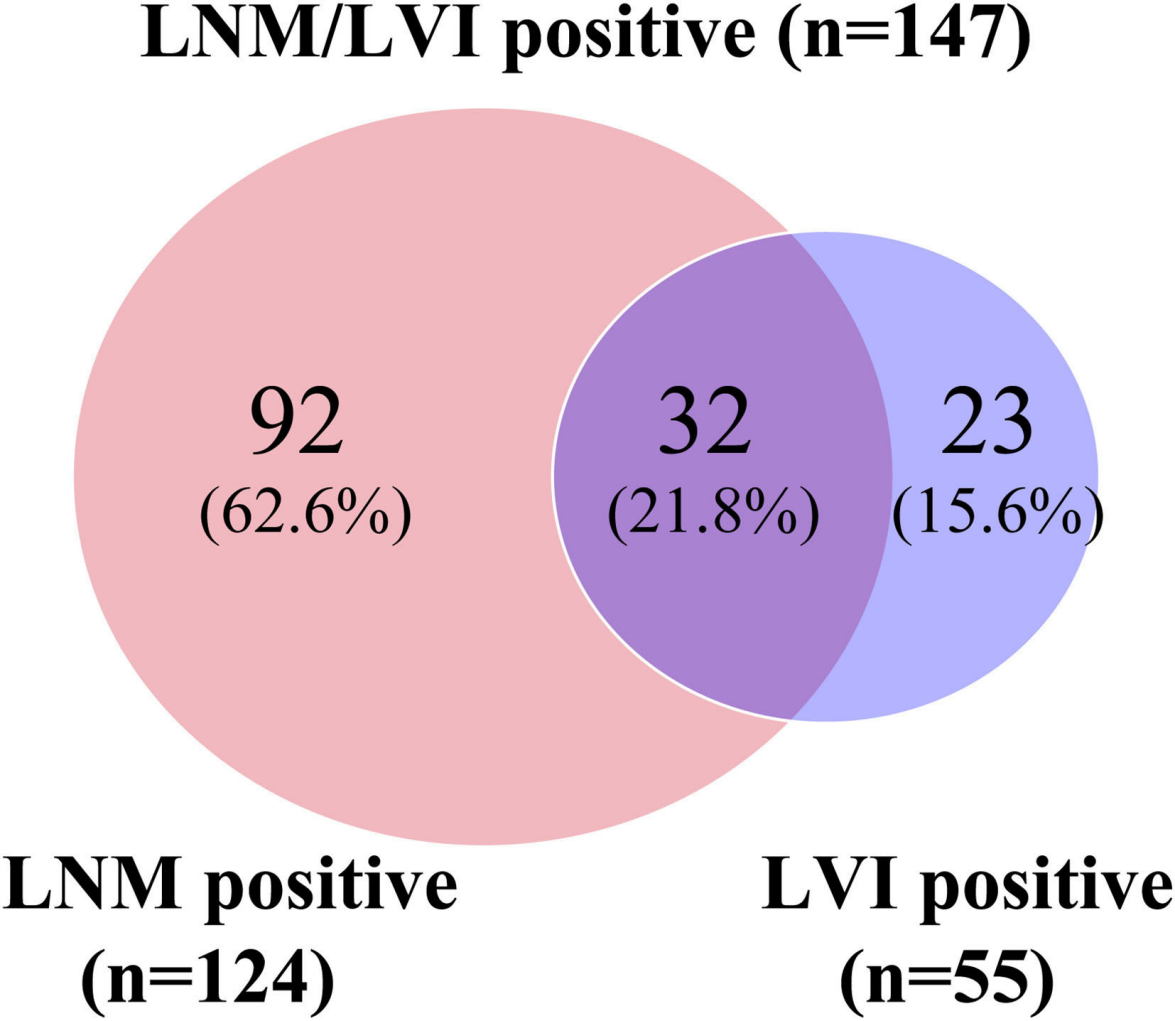
Venn diagram showing details of LNM/LVI. LNM positive and LVI negative (LNM+/LVI–), LNM negative and LVI positive (LNM–/LVI+), and LNM positive and LVI positive (LNM+/LVI+) were regarded as LNM/LVI positive. LNM/LVI negative meant LNM negative and LVI negative (LNM–/LVI–).

In this study, 512 patients with SESCC were enrolled for analysis, with a median age of 61.5 years (range 22–79), including 438 men (85.5%) and 74 women (14.5%). Tumors located in the middle esophagus were found in 361 cases, followed by 133 cases with tumors located in the lower esophagus, and tumors located in the upper esophagus were only found in 18 cases. The median tumor size was 3 cm (range 3–11). A total of 142 patients (27.7%) had tumors confined to the mucosa, and 370 patients (72.3%) had tumors infiltrated into the submucosa. According to the tumor differentiation results, 114 cases (22.3%) were well-differentiated or carcinoma *in situ*, 245 (47.9%) were moderate differentiation, and 153 (29.9%) were poor differentiation. Circumferential extension of lesions in 406 patients (79.3%) was ≤ 1/2 and in 106 patients (20.7%) was >1/2. Among these patients, the macroscopic type of lesions in 271 patients (52.9%) was flat type and in 241 patients (47.1%) was a non-flat type. Only 35 (6.8%) patients had multiple lesions while the other 477 patients (93.2%) did not. There were 147 patients with SESCC who had positive LNM/LVI, of which 92 patients (62.6%) had LNM+/LVI–, 32 patients had LNM+/LVI+ (21.8%), and 23 patients had LNM–/LVI+ (15.6%) (Figure 2), and the remaining 365 patients were an absence of LNM/LVI. The detailed clinicopathological characteristics of 512 patients with SESCC are shown in Table 1.

**Table 1:** Clinicopathological characteristics of patients with SESCC based on LNM/LVI status.

**Variables**	**Total (*n* = 512)**	**LNM/LVI negative (*n* = 365)**	**LNM/LVI positive (*n* = 147)**	***P***
Sex, *n* (%)				0.051
Male	438 (85.5)	305 (83.6)	133 (90.5)	
Female	74 (14.5)	60 (16.4)	14 (9.5)	
Age (years), median (range)	61.5 (22–79)	62 (22–79)	60 (44–78)	0.243
Tumor size (cm), median (range)	3 (1–11)	3 (1–9)	3 (1–11)	**0.002**
Circumferential extension, *n* (%)				0.068
≤ 1/2	406 (79.3)	297 (81.4)	109 (74.1)	
>1/2	106 (20.7)	68 (18.6)	38 (25.9)	
Location within esophagus, *n* (%)				**0.013**
Upper	18 (3.5)	12 (3.3)	6 (4.1)	
Middle	361 (70.5)	271 (74.2)	90 (61.2)	
Lower	133 (26.0)	82 (22.5)	51 (34.7)	
Depth of invasion, *n* (%)				**< 0.001**
Mucosa	142 (27.7)	131 (35.9)	11 (7.5)	
Submucosa	370 (72.3)	234 (64.1)	136 (92.5)	
Tumor differentiation, *n* (%)				**< 0.001**
Well or Carcinoma *in situ*	114 (22.3)	93 (25.5)	21 (14.3)	
Moderate	245 (47.9)	182 (49.9)	63 (42.9)	
Poor	153 (29.9)	90 (24.7)	63 (42.9)	
Macroscopic type, *n* (%)				**< 0.001**
Flat	271 (52.9)	221 (60.5)	50 (34.0)	
Non-flat	241 (47.1)	144 (39.5)	97 (66.0)	
Multiple lesions, *n* (%)				**0.032**
No-	477 (93.2)	346 (94.8)	131 (89.1)	
Yes	35 (6.8)	19 (5.2)	16 (10.9)	

LNM, Lymph node metastasis; LVI, lymphovascular invasion; I, superficial and protruding type; II, flat type; III, superficial and excavated type; P, Categorical variables—χ^2^ test or Fisher's exact test; Continuous variables—Mann–Whitney test.

The bold values mean statistical significance.

### Independent significant factors for LNM/LVI

The clinical characteristics between the LNM/LVI-positive and -negative groups were compared by using univariate analysis, and the results showed that tumor size, tumor location, depth of tumor invasion, tumor differentiation, macroscopic type, and multiple lesions were significantly correlated with LNM/LVI; while age, sex, and circumferential extension were not related with LNM/LVI (Table 1). We also identified the cutoff value of tumor size as 2.5 cm for evaluating LNM/LVI status by using ROC curve analysis (Supplementary Figure S1). Given that the *P*-value of sex between LNM/LVI-positive and -negative groups was 0.051 (very close to 0.05), we then incorporated sex together with tumor size, tumor location, tumor invasion depth, tumor differentiation, macroscopic type, and multiple lesions into the multivariate logistic regression analysis, and found that tumor size, tumor invasion depth, tumor differentiation, and macroscopic type were independent predictive factors of LNM/LVI (Table 2). However, the multivariate logistic analysis showed that the effect of tumor location on LNM/LVI development did not have statistical significance overall (*p* = 0.086); but tumors located in the upper esophagus had a lower risk of LNM/LVI compared with tumors located in the lower esophagus (*p* = 0.028, OR = 0.493). Among these independent predictors, tumor invasion depth and differentiation had the greatest impact on LNM/LVI (*p* < 0.001 in multivariate analysis), suggesting that patients with submucosal tumors which were poorly differentiated had a higher risk for LNM/LVI. The LNM/LVI rates according to the tumor invasion depth and differentiation are summarized in Supplementary Figure S2, Supplementary Table S1.

**Table 2:** Risk factors of LNM/LVI in multivariate logistic analysis.

**Factors**	**β**	**OR**	**95% CI**	***P***
**Sex**
Male	0.538	1.712	0.857–3.422	0.128
Female	Reference			
**Tumor size**
≤ 2.5 cm	Reference			**0.019**
>2.5 cm	0.585	1.795	1.100–2.929	
**Circumferential extension**
≤ 1/2	Reference			
>1/2	0.286	1.332	0.756–2.346	0.321
Tumor location within esophagus				0.086
Upper	−0.707	0.493	0.263–0.926	**0.028**
Middle	−0.363	0.695	0.419–1.155	0.161
Lower	Reference			
**Depth of invasion**
Mucosa	Reference			
Submucosa	1.899	6.681	3.241–13.774	**< 0.001**
Tumor differentiation				**< 0.001**
Well or Carcinoma *in situ*	−1.534	0.216	0.113–0.412	**< 0.001**
Moderate	−1.011	0.364	0.221–0.601	**< 0.001**
Poor	Reference			
**Macroscopic type**
Flat	Reference			
Non-flat	0.652	1.919	1.200–3.069	**0.007**
**Multiple lesions**, ***n*** **(%)**
No	Reference			
Yes	0.438	1.550	0.690–3.481	0.288

LNM, Lymph node metastasis; LVI, lymphovascular invasion; I, superficial and protruding type; II, flat type; III, superficial and excavated type.

The bold values mean statistical significance.

### Identification of predictive factors by LASSO regression

**Figure 3:**
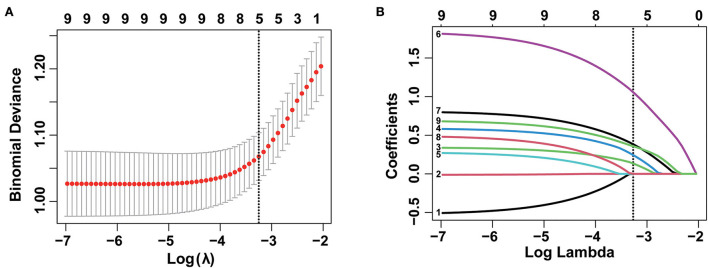
Selection of demographic and clinical features using the least absolute shrinkage and selection operator (LASSO) regression model. **(A)** Selection of optimal parameter (λ) in the LASSO model by using 10-fold cross-validation *via* 1 standard error of the minimum criteria (1-SE criteria). The binomial deviance curve was plotted vs. log(λ). Dotted vertical lines were drawn at the optimal values using the 1-SE criteria. **(B)** LASSO coefficient profiles of nine features. A coefficient profile plot was produced against the log (lambda) sequence. A vertical line was drawn at the value selected using a 10-fold cross-validation, where optimal λ resulted in five non-zero coefficients.

Subsequently, nine variables were incorporated into LASSO regression and five variables including tumor size, depth of invasion, tumor differentiation, tumor location, and macroscopic type were screened out to be associated with LNM/LVI (Figure 3). The weights of each variable affecting LNM/LVI were assessed by calculating the coefficients in the LASSO regression. When λ = 0.039 and log(λ) = −3.251, the specific coefficients of each variable were shown as follows: coefficient of tumor location was 0.130, tumor size was 0.237, tumor invasion depth was 1.046, tumor differentiation was 0.377, and macroscopic type was 0.354 (Supplementary Table S2).

### Confirmation of the best prediction model for LNM/LVI

Four variables (tumor size, tumor invasion depth, tumor differentiation, and macroscopic type) that were confirmed to be significantly associated with LNM/LVI both in multivariate regression and LASSO regression analyses were used to construct a base model (model A). However, for analyzing the predictive effect of tumor location on LNM/LVI, multivariate logistic regression and LASSO regression showed different results. So we then constructed a new model named model B by adding tumor location into model A to explore the significance of tumor location in LNM/LVI prediction. Compared with model A, the AUC of model B for LNM/LVI prediction did not significantly increase, but IDI and cNRI both showed a significant improvement after adding tumor location into model A (Table 3), implying that model B was superior to model A in the prediction of LNM/LVI and tumor location could be considered as a risk factor for LNM/LVI.

**Table 3:** Predictive power comparison of different models in evaluating the status of LNM/LVI.

**Variables**	**Model A (base model)**	**Model B**	***P***
AUC (95%CI)	0.763 (0.724–0.799)	0.776 (0.738–0.812)	0.077
IDI (95%CI)	Reference	0.016 (0.004–0.028)	**0.007**
cNRI (95%CI)	Reference	0.308 (0.119–0.497)	**0.001**

Model A, Tumor size + Depth of invasion + Tumor differentiation + Macroscopic type; Model B, Model A + Tumor location; AUC, area under curve; IDI, integrated discrimination improvement; cNRI, continuous net reclassification improvement.

The bold values mean statistical significance.

### Development and validation of an LNM/LVI-predicting nomogram and its score system

**Figure 4:**
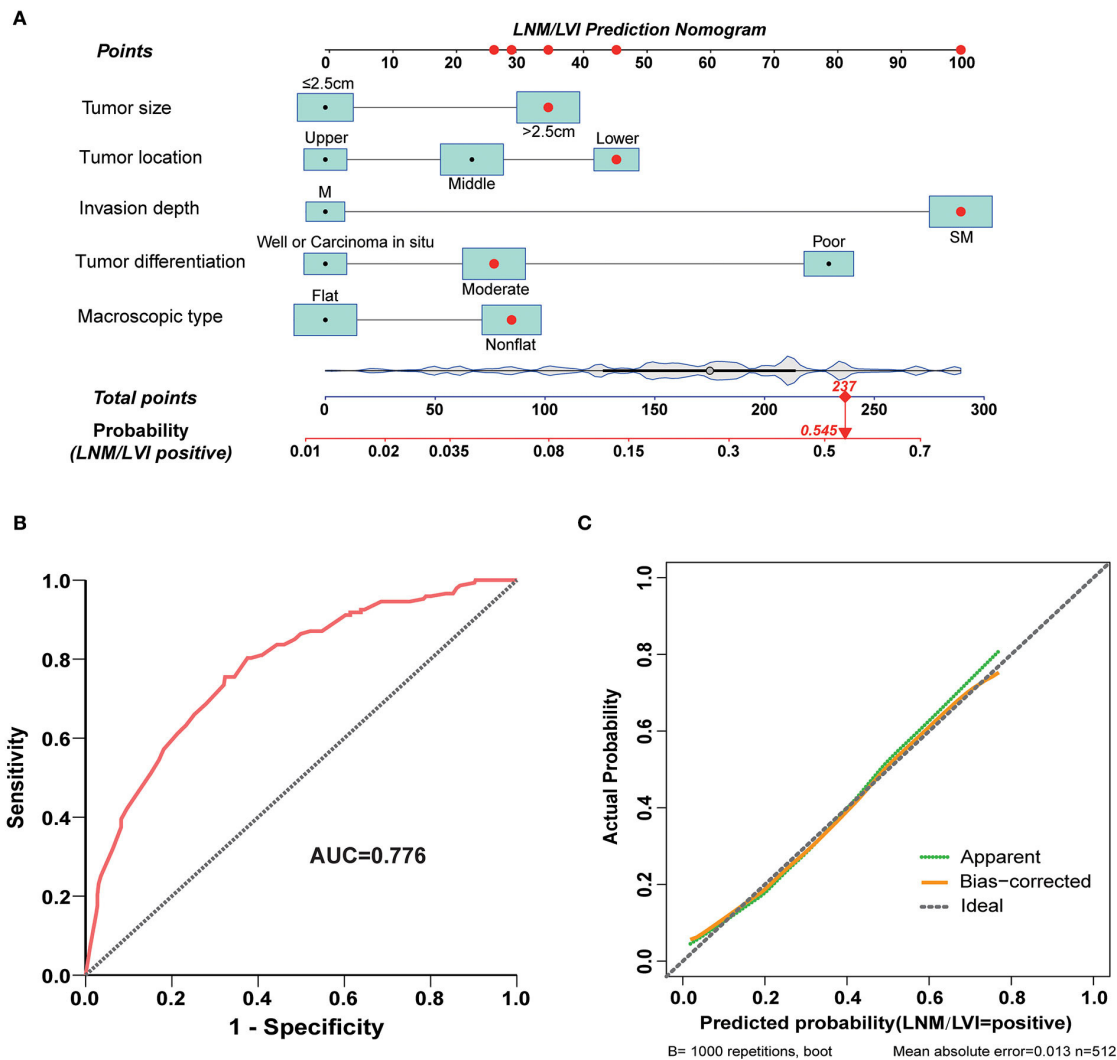
A nomogram for predicting LNM/LVI and its predictive performance. **(A)** The nomogram for predicting LNM/LVI was constructed by incorporating tumor size, tumor invasion depth, tumor differentiation, tumor location, and macroscopic type. Locate the patient's characteristic on a variable row and draw a vertical line straight up to the points' row (top) to assign a point value for the variable. Adding up the total number of points and dropping a vertical line from the total points' row to obtain the probability of a predictive outcome. **(B)** The predictive ability of this nomogram was demonstrated by a ROC curve with an AUC of 0.776. **(C)** Calibration curve of the nomogram for predicting LNM/LVI in SESCC, based on internal validation with a bootstrap resampling frequency of 1,000.

Next, we used the five variables included in model B (tumor size, tumor invasion depth, tumor differentiation, macroscopic type, and tumor location) to develop a nomogram that could predict the probability of LNM/LVI presence (Figure 4A). ROC analysis showed that the AUC of this nomogram in LNM/LVI prediction was 0.776 (Figure 4B), suggesting the nomogram had a good predictive performance for LNM/LVI. In addition, the calibration curve demonstrated that the nomogram-predicted LNM/LVI status was nearly consistent with the actual LNM/LVI status (Figure 4C). Each variable in the nomogram was assigned a corresponding risk score (the detailed scores of these five variables are seen in Supplementary Table S3). The scores of each variable in this nomogram were summed to obtain the total scores ranging from 0 to 289. The optimal cut-off value of the total scores was identified as 179 based on the maximum Youden index in ROC analysis (Supplementary Table S4).

### Clinical use of the nomogram for LNM/LVI prediction

**Figure 5:**
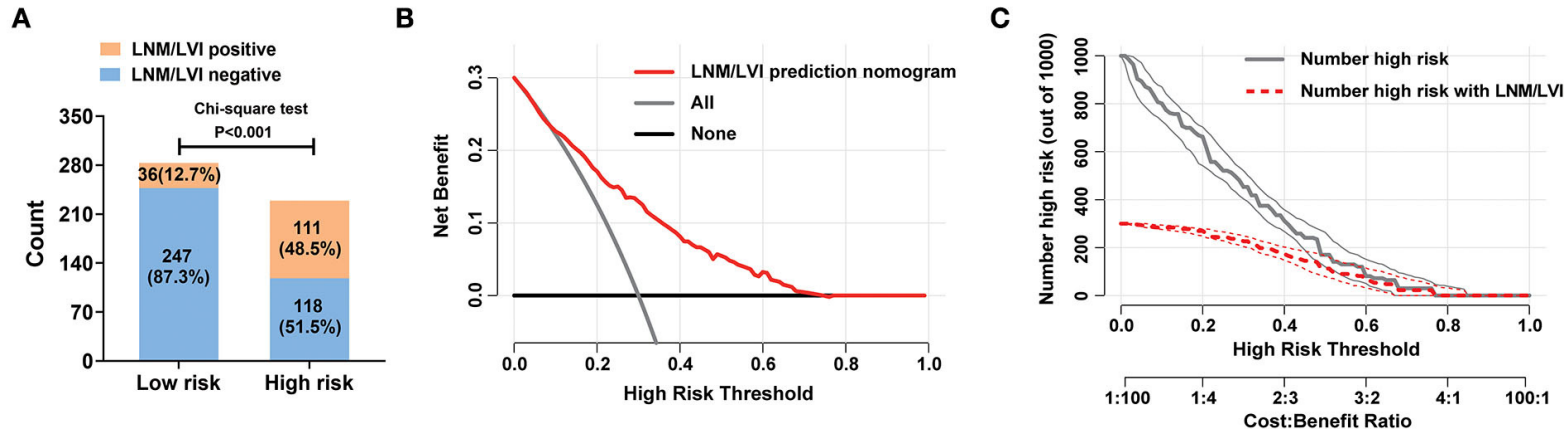
Clinical utility of the nomogram for predicting LNM/LVI in SESCC. **(A)** The discriminatory power of the nomogram for LNM/LVI was shown with a bar chart. Risk classification of the predictive nomograms was conducted by the maximum Youden index in a ROC analysis, and the performance in distinguishing the LNM/LVI was confirmed by the chi-square test. **(B)** Decision curve of the nomograms for predicting the presence of LNM/LVI. The horizontal solid black line represents the hypothesis that no patients experienced the presence of MVI or its M2 grade, and the solid gray line represents the hypothesis that all patients met the endpoint. **(C)** Clinical impact curve of the nomogram for predicting the status of LNM/LVI. At different threshold probabilities within a given population, the number of high-risk patients and the number of high-risk patients with LNM/LVI can be known from the *y*-axis.

According to the cut-off value determined above, patients with SESCC were divided into LNM/LVI high-risk (total score >179) and low-risk (total score ≤ 179) groups. The ratio of positive LNM/LVI in the high-risk group was 48.5%, while only 12.7% of patients with SESCC presented with LNM/LVI in the low-risk group, which meant that patients with SESCC in the high-risk group had a significantly higher probability of LNM/LVI (Figure 5A). Then, the DCA results clarified that taking treatment based on the LNM/LVI nomogram predicted results had a higher net benefit than treating all or zero patients when the risk threshold of a patient was >0.2 (Figure 5B), suggesting that the clinical utility of this nomogram was good. In addition, we plotted a clinical impact curve of the nomogram for predicting LNM/LVI to gain a more comprehensive understanding of the clinical significance of our nomogram (Figure 5C). It can be seen from this figure that the distance between the predicted number of high risk (the gray curve) and the actual number of high risk with LNM/LVI (the red curve) approaches as the risk threshold increases, indicating a good predictive power of this nomogram.

### Random forest model and its predictive effectiveness for LNM/LVI

**Figure 6:**
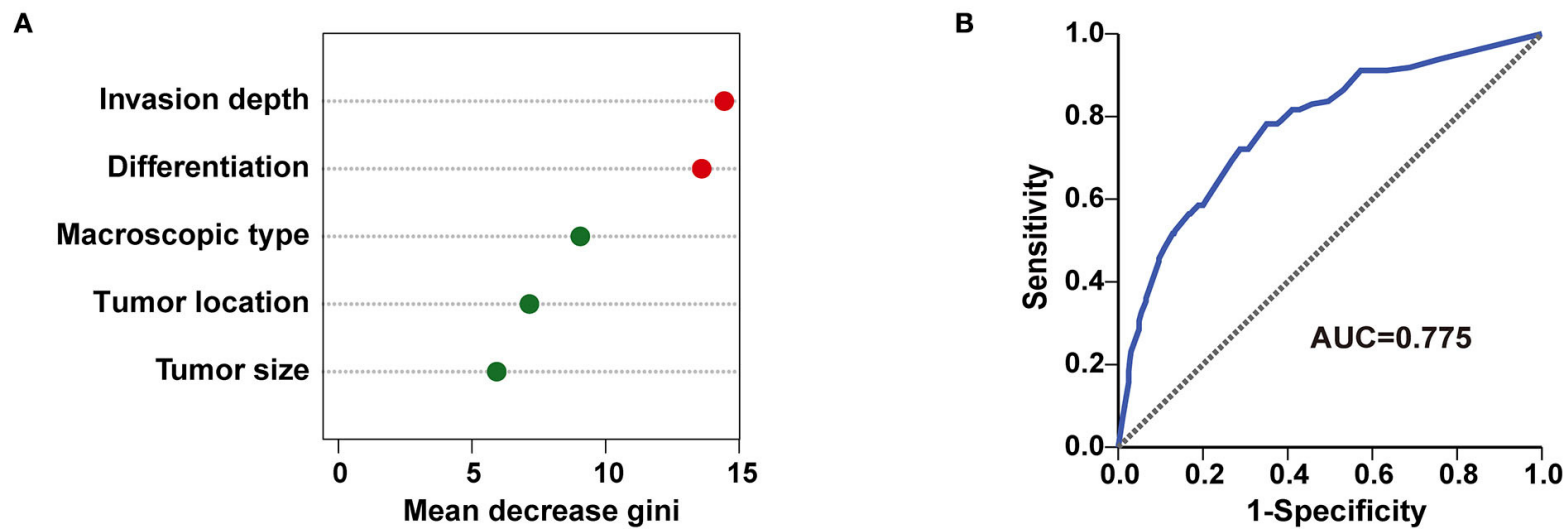
A random forest model for predicting LNM/LVI. **(A)** The importance ranking of factors related to LNM/LVI in SESCC, including tumor size, tumor invasion depth, tumor differentiation, tumor location, and macroscopic type. The larger the mean decrease of Gini, the more important the factor was. **(B)** The predictive performance of the random forest model (incorporated tumor size, tumor invasion depth, tumor differentiation, tumor location, and macroscopic type) was demonstrated by a ROC curve with an AUC of 0.775.

Furthermore, all 512 samples in our study were used to construct a random forest model. The five independent risk factors identified above related to LNM/LVI in SESCC, including tumor size, tumor invasion depth, tumor differentiation, macroscopic type, and tumor location, were ranked according to the mean decreased Gini (Figure 6A). Tumor invasion depth and tumor differentiation were the two most important variables in the random forest model because their mean decreased Gini was higher than other variables. The out of bage (OOB) error rate was 25.98% when the number of the decision tree was 500 (Supplementary Figure S3), meaning the prediction performance of this random forest model was not bad. ROC analysis was adopted to further assess the efficacy of the random forest model, and the AUC of this model for predicting LNM/LVI in SESCC was 0.775 (Figure 6B), which was similar to the AUC (0.776) of LNM/LVI-predicting nomogram (Figure 4B). Finally, we chose the nomogram as a clinical tool for LNM/LVI prediction in this study due to its supply of detailed scores for each risk factor.

## Discussion

Endoscopic resection (ER) represented by EMR or ESD has been widely carried out to treat early esophageal cancer because it causes less trauma and improves the postoperative quality of life compared with traditional esophagectomy (27, 28). Nevertheless, ER is unable to dissect the lymph nodes around the esophagus with potential tumor metastasis (29). Also, there is a lack of accurate methods to preoperatively assess the status of LNM in esophageal cancer (30). If SESCC patients with potential LNM undergo endoscopic resection, they will face the risk of tumor postoperative recurrence and metastasis (7, 29). Hence, ER is suitable for SESCC patients with negative LNM, and exploring the preoperative risk factors of LNM is important to select the best treatment strategy for patients with SESCC.

Some studies have demonstrated a strong correlation between LVI and LNM (31, 32), and 48.7% (18/37) of LNM-positive patients with SESCC have LVI based on the results of hematoxylin-eosin staining (33). In this study, 25.8% (32/124) of LNM-positive patients with SESCC were presented with LVI, while LVI-positive cases were only found to account for 5.9% (23/388) of LNM-negative patients with SESCC. LVI was also considered to be an independent risk factor for the development of LNM and led to a poor prognosis for patients with esophageal cancer (14, 16). Based on these viewpoints, LVI has great clinical guiding significance for the selection of treatment strategies in patients with SESCC. More importantly, LVI is difficult to be diagnosed before surgery and is mostly identified by postoperative pathological examination (17). However, prior studies on LNM in patients with SESCC included LVI as one of the predictive risk factors in statistical analysis (31–34), which may greatly limit the clinical application of the findings from these studies. Therefore, in this study, we combined LNM and LVI as a unitary outcome and named it LNM/LVI to evaluate the status of LNM and LVI more reasonably and comprehensively, and further investigated the preoperative independent risk factors associated with LNM/LVI to provide more precise evidence for the selection of endoscopic treatment in patients with SESCC.

Tumor size, depth of invasion, and tumor differentiation had been confirmed as independent risk factors for LNM of SESCC in most of the previous studies (31, 32, 34, 35). Therefore, it is speculated that these three variables may also be correlated with the development of LNM/LVI in SESCC. Then, our study identified that tumor size was significantly associated with LNM/LVI in patients with SESCC. The risk of LNM/LVI was also different between mucosal and submucosal cancers in this study. Compared with mucosal cancers, SESCC with submucosal infiltration was more likely to have LNM/LVI (the odds ratio was 6.681, Table 2). Only 7.8% (11/142) of patients with SESCC confined to the mucosa were in presence of LNM/LVI, whereas the percentage of LNM/LVI positive cases increased dramatically to 36.8% (136/370) in patients with SESCC whose tumors invaded into the submucosa (Table 1). As per our speculation, tumor histological differentiation was also found as a significant risk factor for LNM/LVI in our study. In submucosal cancers, 5.14% of SESCC with good differentiation presented with LNM/LVI, which is relatively safe for receiving ER. Moreover, LNM/LVI appeared in 15.68% of moderate differentiated submucosal SESCC and 15.95% of poor differentiated submucosal SESCC (Supplementary Figure S2, the detailed LNM/LVI status according to invasion depth and tumor differentiation is shown in Supplementary Table S1), suggesting that these patients need to be treated with lymph node dissection.

Interestingly, the macroscopic type of SESCC was also identified as a significant risk factor for the LNM/LVI of SESCC in this study. The macroscopic type of esophageal cancer had been reported to be correlated with the depth of tumor invasion, which might affect the status of LNM/LVI (36, 37). Furthermore, in our multivariate logistic analysis for the risk factors of LNM/LVI, the variable tumor location did not show any overall statistical significance (*p* = 0.086); on the contrary, in LASSO regression analysis, the location of SESCC was demonstrated to be associated with LNM/LVI (Supplementary Table S2). Since both multivariate logistic and LASSO regression analyses revealed that tumor size, tumor invasion depth, tumor differentiation, and macroscopic type were significant risk factors for LNM/LVI, these four variables were used to establish a base model (model A). We then built a new model named model B by adding tumor location to model A for exploring whether tumor location was a risk factor of LNM/LVI in SESCC. Compared with model A, model B including tumor location exhibited a remarkable improvement in LNM/LVI prediction according to the results of IDI and cNRI analyses (Table 3), implying that tumor location within the esophagus was a true risk factor for LNM/LVI. The impact of tumor location on LNM/LVI may be attributed to the difference in the number of regional lymph nodes around different locations of the esophagus, especially the regional lymph nodes around the lower esophagus are more abundant than those around the upper and middle esophagus (29), which may make the lower esophageal tumor more likely to develop LNM/LVI.

Nomogram has been regarded as a useful prediction tool with a supply of risk scores for each patient and is widely used in the prediction of prognosis or outcome events (38, 39). In this study, we established a nomogram for predicting LNM/LVI with high accuracy by incorporating five variables such as tumor size, depth of tumor invasion, tumor differentiation, tumor location, and macroscopic type. Satisfactory predictive performance of this nomogram was confirmed by an AUC of 0.776. The calibration curve also suggested that the predicted results of the nomogram were in excellent agreement with the actual observed events. An optimal cut-off value of the total scores derived from our nomogram was then determined to be 179 based on the maximum Youden index in ROC analysis. Patients with a total score of >179 or ≤ 179 were assigned to a high-risk or low-risk group of LNM/LVI. Esophagectomy plus lymph node dissection will be recommended if a patient with SESCC belongs to a high-risk group. Additionally, the good clinical utility and predictive power of our nomogram for predicting LNM/LVI were demonstrated by plotting the decision curve and clinical impact curve in DCA.

To further evaluate the impact of the five variables (tumor size, tumor invasion depth, tumor differentiation, tumor location, and macroscopic type) in this nomogram on LNM/LVI, we incorporated them into the random forest model and calculated the importance of each variable to LNM/LVI. The results showed that the random forest model can well-distinguish the positive LNM/LVI and negative LNM/LVI in patients with SESCC, with an AUC of 0.775 in LNM/LVI prediction. Because the established nonogram above assigned a detailed risk score to each patient, and its predictive performance (AUC was 0.776) for LNM/LVI was similar to that of the random forest model, we finally used the nomogram as a discriminant tool to predict LNM/LVI of patients with SESCC in this study.

A majority of studies on clinical prediction models usually split the dataset into a training set and validation set, but our data were not analyzed in this way attributed to the limited sample size of the current study. Besides, this approach does not make full utilization of all available data to establish a prediction model, which may result in statistical inefficiency or even a waste of data (40). Unfortunately, there are still several limitations in this study. First, the clinical data of this study were collected retrospectively, and data selection bias was inevitable. Thus, it is necessary to carry out a prospective study with balanced patients' characteristics to deeply affirm the reliability and feasibility of our nomogram in the future. Second, the tumor size and tumor invasion depth recorded in this study were determined by postoperative pathological findings, but we took it as preoperative data in statistical analysis. Although it is possible to obtain a relatively accurate tumor size and tumor invasion depth by endoscopy and endoscopic ultrasonography before surgery, a certain deviation may exist when compared with the postoperative pathological results. Third, the patients enrolled in this study were all from a single institution, and the predictive performance of our nomogram was also not validated. Therefore, a multicenter study should be conducted to validate our results. Finally, this nomogram was established only by using limited clinical variables, and some serological markers associated with LNM/LVI also need to be identified and incorporated into the nomogram to further advance its prediction accuracy.

In summary, to our knowledge, this is the first study to combine the LNM and LVI as a unitary outcome and investigate the impact of preoperative factors on potential LNM/LVI in patients with SESCC. We demonstrated that tumor size, tumor invasion depth, tumor differentiation, tumor location, and macroscopic type were significant risk factors for the presence of LNM/LVI in patients with SESCC. Then, a nomogram and a random forest model for predicting LNM/LVI were established by incorporating these five variables. The good clinical utility of our nomogram was confirmed by DCA, and the risk score system also made the nomogram more clinically useful than the random forest model despite their similar AUC. Hence, this nomogram was adopted as a final tool for LNM/LVI prediction in patients with SESCC. According to the scoring system of our nomogram, if a patient with SESCC is classified as high risk for LNM/LVI, esophagectomy plus lymph node dissection will be prioritized to reduce potential tumor recurrence in the future. Last but not least, the nomogram established in this study is a user-friendly and practical tool to facilitate optimal treatment strategy selection for patients with SESCC by making an individualized LNM/LVI prediction.

## Data availability statement

The raw data supporting the conclusions of this article will be made available by the authors, without undue reservation.

## Ethics statement

This retrospective study was approved by the Ethics Committee of the Zhejiang Cancer Hospital. Written informed consent for participation was not required for this study in accordance with the National Legislation and the Institutional requirements.

## Author contributions

SW and RR conceived the idea, designed the study, and revised the manuscript. JY and QS collected data. YT and SC analyzed the data and drafted the manuscript. All authors contributed to the article and approved the submitted version.

## Funding

This study was funded by the Medical Health Science and Technology Project of Zhejiang Province (No. 2022KY619).

## Conflict of interest

The authors declare that the research was conducted in the absence of any commercial or financial relationships that could be construed as a potential conflict of interest.

## Publisher's note

All claims expressed in this article are solely those of the authors and do not necessarily represent those of their affiliated organizations, or those of the publisher, the editors and the reviewers. Any product that may be evaluated in this article, or claim that may be made by its manufacturer, is not guaranteed or endorsed by the publisher.

## References

[1] Sung H Ferlay J Siegel RL Laversanne M Soerjomataram I Jemal A. Global cancer statistics 2020: globocan estimates of incidence and mortality worldwide for 36 cancers in 185 countries. CA Cancer J Clin. (2021) 71:209–49. 10.3322/caac.21660 33538338

[2] Chen W Zheng R Baade PD Zhang S Zeng H Bray F. Cancer statistics in china, 2015. CA Cancer J Clin. (2016) 66:115–32. 10.3322/caac.21338 26808342

[3] Jacobs M Macefield RC Elbers RG Sitnikova K Korfage IJ Smets EM. Meta-analysis shows clinically relevant and long-lasting deterioration in health-related quality of life after esophageal cancer surgery. Qual Life Res. (2014) 23:1097–115. 10.1007/s11136-013-0576-5 24129668

[4] Ishihara R Arima M Iizuka T Oyama T Katada C Kato M. Endoscopic submucosal dissection/endoscopic mucosal resection guidelines for esophageal cancer. Dig Endosc. (2020) 32:452–93. 10.1111/den.13654 32072683

[5] Sharma P Shaheen NJ Katzka D Bergman J. Aga clinical practice update on endoscopic treatment of barrett's esophagus with dysplasia and/or early cancer: expert review. Gastroenterology. (2020) 158:760–9. 10.1053/j.gastro.2019.09.051 31730766

[6] Berger A Rahmi G Perrod G Pioche M Canard JM Cesbron-Metivier E. Long-term follow-up after endoscopic resection for superficial esophageal squamous cell carcinoma: a multicenter western study. Endoscopy. (2019) 51:298–306. 10.1055/a-0732-5317 30261535

[7] Kuwano H Nishimura Y Oyama T Kato H Kitagawa Y Kusano M. Guidelines for diagnosis and treatment of carcinoma of the esophagus april 2012 edited by the japan esophageal society. Esophagus. (2015) 12:1–30. 10.1007/s10388-014-0465-1 25620903 PMC4297610

[8] Rice TW Ishwaran H Hofstetter WL Schipper PH Kesler KA Law S. Esophageal cancer: associations with (pn+) lymph node metastases. Ann Surg. (2017) 265:122–9. 10.1097/SLA.0000000000001594 28009736 PMC5405457

[9] Lightdale CJ Kulkarni KG. Role of endoscopic ultrasonography in the staging and follow-up of esophageal cancer. J Clin Oncol. (2005) 23:4483–9. 10.1200/JCO.2005.20.644 16002838

[10] Kim K Park SJ Kim BT Lee KS Shim YM. Evaluation of lymph node metastases in squamous cell carcinoma of the esophagus with positron emission tomography. Ann Thorac Surg. (2001) 71:290–4. 10.1016/S0003-4975(00)02221-9 11216764

[11] Yoon YC Lee KS Shim YM Kim BT Kim K Kim TS. Metastasis to regional lymph nodes in patients with esophageal squamous cell carcinoma: ct versus fdg pet for presurgical detection prospective study. Radiology. (2003) 227:764–70. 10.1148/radiol.2281020423 12773680

[12] Choi J Kim SG Kim JS Jung HC Song IS. Comparison of endoscopic ultrasonography (eus), positron emission tomography (pet), and computed tomography (ct) in the preoperative locoregional staging of resectable esophageal cancer. Surg Endosc. (2010) 24:1380–6. 10.1007/s00464-009-0783-x 20033712

[13] Hogan J Chang KH Duff G Samaha G Kelly N Burton M. Lymphovascular invasion: a comprehensive appraisal in colon and rectal adenocarcinoma. Dis Colon Rectum. (2015) 58:547–55. 10.1097/DCR.0000000000000361 25944426

[14] Schiefer AI Schoppmann SF Birner P. Lymphovascular invasion of tumor cells in lymph node metastases has a negative impact on survival in esophageal cancer. Surgery. (2016) 160:331–40. 10.1016/j.surg.2016.02.034 27085684

[15] Yu JW Wu JG Tajima Y Li XQ Du GY Zheng LH. Study on lymph node metastasis correlated to lymphangiogenesis, lymphatic vessel invasion, and lymph node micrometastasis in gastric cancer. J Surg Res. (2011) 168:188–96. 10.1016/j.jss.2009.10.030 20189585

[16] Wang Z Chen P Wang F Lin L Liu S. Lymphovascular invasion as an independent prognostic indicator in radically resected thoracic esophageal squamous cell carcinoma. Thorac Cancer. (2019) 10:150–5. 10.1111/1759-7714.12922 30484962 PMC6360202

[17] Chen W Wang Y Bai G Hu C. Can lymphovascular invasion be predicted by preoperative contrast-enhanced ct in esophageal squamous cell carcinoma? Technol Cancer Res Treat. (2022) 21:1180452312. 10.1177/15330338221111229 35790460 PMC9340382

[18] Ma Z Liang C Huang Y He L Liang C Chen X. Can lymphovascular invasion be predicted by preoperative multiphasic dynamic ct in patients with advanced gastric cancer? Eur Radiol. (2017) 27:3383–91. 10.1007/s00330-016-4695-6 27999983

[19] Sauerbrei W Royston P Binder H. Selection of important variables and determination of functional form for continuous predictors in multivariable model building. Stat Med. (2007) 26:5512–28. 10.1002/sim.3148 18058845

[20] Pencina MJ D'Agostino RS Steyerberg EW. Extensions of net reclassification improvement calculations to measure usefulness of new biomarkers. Stat Med. (2011) 30:11–21. 10.1002/sim.4085 21204120 PMC3341973

[21] Pencina MJ D'Agostino RS D'Agostino RJ Vasan RS. Evaluating the added predictive ability of a new marker: from area under the roc curve to reclassification and beyond. Stat Med. (2008) 27:157–72, 207–12. 10.1002/sim.2929 17569110

[22] Cho CS Gonen M Shia J Kattan MW Klimstra DS Jarnagin WR. A novel prognostic nomogram is more accurate than conventional staging systems for predicting survival after resection of hepatocellular carcinoma. J Am Coll Surg. (2008) 206:281–91. 10.1016/j.jamcollsurg.2007.07.031 18222381

[23] Vickers AJ Cronin AM Elkin EB Gonen M. Extensions to decision curve analysis, a novel method for evaluating diagnostic tests, prediction models and molecular markers. BMC Med Inform Decis Mak. (2008) 8:53. 10.1186/1472-6947-8-53 19036144 PMC2611975

[24] Amaratunga D Cabrera J Lee YS. Enriched random forests. Bioinformatics. (2008) 24:2010–4. 10.1093/bioinformatics/btn356 18650208

[25] Pavey TG Gilson ND Gomersall SR Clark B Trost SG. Field evaluation of a random forest activity classifier for wrist-worn accelerometer data. J Sci Med Sport. (2017) 20:75–80. 10.1016/j.jsams.2016.06.003 27372275

[26] Sauer CM Sasson D Paik KE McCague N Celi LA Sanchez FI. Feature selection and prediction of treatment failure in tuberculosis. PLoS ONE. (2018) 13:e207491. 10.1371/journal.pone.0207491 30458029 PMC6245785

[27] Ishihara R Iishi H Uedo N Takeuchi Y Yamamoto S Yamada T. Comparison of emr and endoscopic submucosal dissection for en bloc resection of early esophageal cancers in japan. Gastrointest Endosc. (2008) 68:1066–72. 10.1016/j.gie.2008.03.1114 18620345

[28] Shi Q Zhong YS Yao LQ Zhou PH Xu MD Wang P. Endoscopic submucosal dissection for treatment of esophageal submucosal tumors originating from the muscularis propria layer. Gastrointest Endosc. (2011) 74:1194–200. 10.1016/j.gie.2011.07.039 21963065

[29] Japan Esophageal Society office@ esophagus. jp. Japanese classification of esophageal cancer, 11th edition: part I. Esophagus. (2017) 14:1–36. 10.1007/s10388-016-0551-7 28111535 PMC5222932

[30] Semenkovich TR Yan Y Subramanian M Meyers BF Kozower BD Nava R. A clinical nomogram for predicting node-positive disease in esophageal cancer. Ann Surg. (2021) 273:e214–21. 10.1097/SLA.0000000000003450 31274650 PMC6940556

[31] Min BH Yang JW Min YW Baek SY Kim S Kim HK. Nomogram for prediction of lymph node metastasis in patients with superficial esophageal squamous cell carcinoma. J Gastroen Hepatol. (2020) 35:1009–15. 10.1111/jgh.14915 31674067

[32] Ma DW Jung DH Kim J Park JJ Youn YH Park H. Predicting lymph node metastasis for endoscopic resection of superficial esophageal squamous cell carcinoma. J Thorac Cardiovasc Surg. (2019) 157:397–402. 10.1016/j.jtcvs.2018.07.034 30143376

[33] Mitobe J Ikegami M Urashima M Takahashi H Goda K Tajiri H. Clinicopathological investigation of lymph node metastasis predictors in superficial esophageal squamous cell carcinoma with a focus on evaluation of lympho-vascular invasion. Scand J Gastroentero. (2013) 48:1173–82. 10.3109/00365521.2013.832365 23971724

[34] Li B Chen H Xiang J Zhang Y Kong Y Garfield DH. Prevalence of lymph node metastases in superficial esophageal squamous cell carcinoma. J Thorac Cardiovasc Surg. (2013) 146:1198–203. 10.1016/j.jtcvs.2013.07.006 23988285

[35] Zhou Y Du J Li H Luo J Chen L Wang W. Clinicopathologic analysis of lymph node status in superficial esophageal squamous carcinoma. World J Surg Oncol. (2016) 14:1–7. 10.1186/s12957-016-1016-0 27729036 PMC5059900

[36] Update on the paris classification of superficial neoplastic lesions in the digestive tract. Endoscopy. (2005) 37:570–8. 10.1055/s-2005-861352 15933932

[37] Jia R Luan Q Wang J Hou D Zhao S. Analysis of predictors for lymph node metastasis in patients with superficial esophageal carcinoma. Gastroent Res Pract. (2016) 2016:1–6. 10.1155/2016/3797615 27799939 PMC5069363

[38] Iasonos A Schrag D Raj GV Panageas KS. How to build and interpret a nomogram for cancer prognosis. J Clin Oncol. (2008) 26:1364–70. 10.1200/JCO.2007.12.9791 18323559

[39] Shariat SF Capitanio U Jeldres C Karakiewicz PI. Can nomograms be superior to other prediction tools? BJU Int. (2009) 103:492–5, 495–7. 10.1111/j.1464-410X.2008.08073.x 18990135

[40] Moons KG Kengne AP Woodward M Royston P Vergouwe Y Altman DG. Risk prediction models: I. Development, internal validation, and assessing the incremental value of a new (bio)marker. Heart. (2012) 98:683–90. 10.1136/heartjnl-2011-301246 22397945

