# Supplementary material for: Risk factors of lymph node metastasis or lymphovascular invasion for superficial esophageal squamous cell carcinoma: A practical and effective predictive nomogram based on a cancer hospital data
Source: Front Med (Lausanne). 2022 Nov 17;9:1038097. doi: 10.3389/fmed.2022.1038097 (PMC9713002; doi:10.3389/fmed.2022.1038097)
Supplement: Supplementary file 1 [file Data_Sheet_1.pdf]

Risk factors of lymph node metastasis or lymphovascular invasion for superficial esophageal squamous cell carcinoma: a practical and effective predictive nomogram based on a cancer hospital data

Yali Tao†, Shengsen Chen†, Jiangping Yu, Qiwen Shen, Rongwei Ruan*, Shi Wang*

Department of Endoscopy, Cancer Hospital of the University of Chinese Academy of Sciences (Zhejiang Cancer Hospital), Institute of Basic Medicine and Cancer (IBMC), Chinese Academy of Sciences, Hangzhou 310022, Zhejiang, China.

*Corresponding author:
Shi Wang, E-mail: wangshi@zjcc.org.cn. Rongwei Ruan, Email: ruanrw2006@163.com.

†These authors have contributed equally to this work

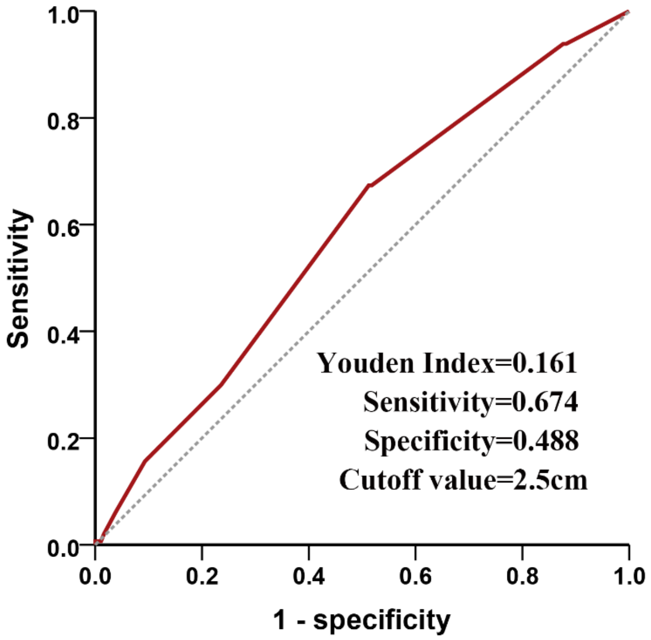

Figure S1. Determination of the optimal cutoff value for tumor size based on the ROC analysis in training set.

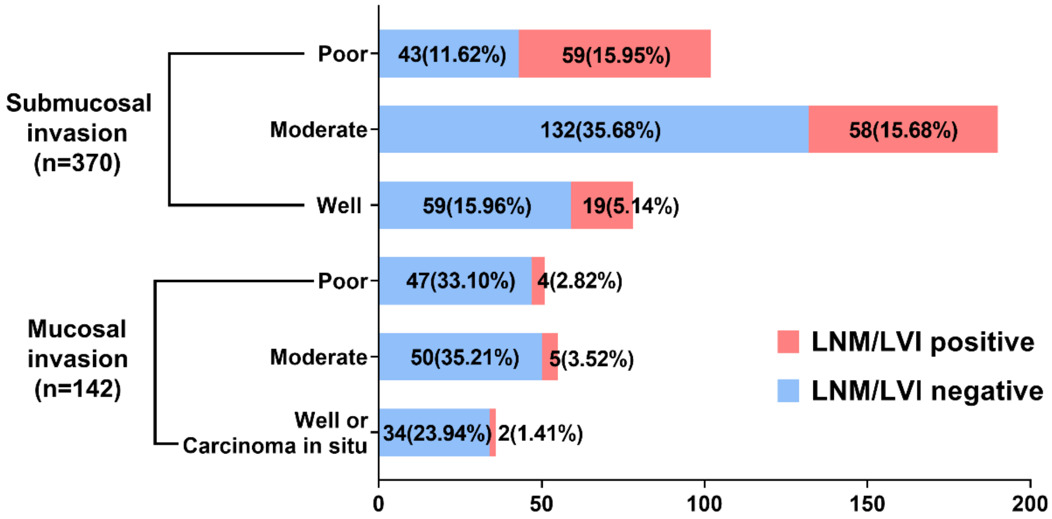

Figure S2. Distribution of LNM/LVI status in esophageal squamous cell carcinoma patients based on invasion depth and tumor differentiation.

**Table S1. LNM/LVI detailed status according to tumor invasion depth and tumor differentiation.**				
	LNM (-) LVI(+), n (%)	LNM(+)LVI(-), n(%)	LNM(+)LVI(+), n(%)	LNM(-)LVI(-), n(%)
Mucosal invasion (n=142)				
Well or Carcinoma in situ	0(0)	2(1.408)	0(0)	34(23.944)
Moderate	0(0)	3(2.113)	2(1.408)	50(35.211)
Poor	0(0)	3(2.113)	1(0.704)	47(33.099)
Submucosal invasion(n=370)				
Well	2(0.541)	12(3.243)	5(1.351)	59(15.946)
Moderate	11(2.973)	37(10.000)	10(2.703)	132(35.676)
Poor	10(2.703)	35(9.459)	14(3.784)	43(11.622)

**Table S2. Coefficients for selected features in the LASSO regression models on LNM/LVI status.**	
$\lambda_{1\text{-SE}}$= 0.03875024, log(λ)= -3.250618	
Index	coefficient
Tumor location	0.1297490
Tumor size	0.2370556
Depth of invasion	1.0464772
Tumor differentiation	0.3771133
Macroscopic type	0.3544843

**Table S3. Point assignments and predictive scores for each variable in the nomogram model.**	
Variables	Nomogram score
	LNM/LVI
Tumor size (cm)	
≤2.5	0
>2.5	35
Tumor location within esophagus	
Upper	0
Middle	23
Lower	46
Depth of invasion	
Mucosa	0
Submucosa	100
Tumor differentiation	
Well or Carcinoma in situ	0
Moderate	27
Poor	79
Macroscopic type	
Flat	0
Nonflat	29
NA, not available	

Table S4. Identification of the optimal cutoff value of the total nomogram scores for LNM/LVI prediction in ROC curve.

Criterion (Total scores)	Sensitivity%	95% CI	Specificity%	95% CI	Youden Index
≥0	100.00	97.5 - 100.0	0.00	0.0 - 1.0	0
>0	100.00	97.5 - 100.0	0.55	0.07 - 2.0	0.0055
>23	100.00	97.5 - 100.0	2.47	1.1 - 4.6	0.0247
>27	100.00	97.5 - 100.0	3.84	2.1 - 6.4	0.0384
>35	100.00	97.5 - 100.0	4.66	2.7 - 7.4	0.0466
>46	100.00	97.5 - 100.0	5.21	3.2 - 8.0	0.0521
>50	100.00	97.5 - 100.0	9.59	6.8 - 13.1	0.0959
>52	99.32	96.3 - 100.0	9.86	7.0 - 13.4	0.0918
>58	98.64	95.2 - 99.8	13.15	9.9 - 17.1	0.1179
>62	97.96	94.2 - 99.6	13.97	10.6 - 18.0	0.1193
>73	96.60	92.2 - 98.9	14.79	11.3 - 18.9	0.1139
>75	96.60	92.2 - 98.9	15.07	11.6 - 19.2	0.1167
>79	96.60	92.2 - 98.9	15.89	12.3 - 20.1	0.1249
>81	96.60	92.2 - 98.9	16.44	12.8 - 20.6	0.1304
>85	95.92	91.3 - 98.5	20.00	16.0 - 24.5	0.1592
>87	95.92	91.3 - 98.5	20.82	16.8 - 25.4	0.1674
>91	95.92	91.3 - 98.5	21.10	17.0 - 25.6	0.1702
>100	95.24	90.4 - 98.1	21.64	17.5 - 26.2	0.1688
>102	94.56	89.6 - 97.6	24.93	20.6 - 29.7	0.1949
>108	94.56	89.6 - 97.6	27.40	22.9 - 32.3	0.2196
>110	94.56	89.6 - 97.6	27.67	23.1 - 32.6	0.2223
>114	94.56	89.6 - 97.6	29.32	24.7 - 34.3	0.2388
>123	94.56	89.6 - 97.6	30.41	25.7 - 35.4	0.2497
>125	94.56	89.6 - 97.6	31.51	26.8 - 36.5	0.2607
>127	92.52	87.0 - 96.2	35.34	30.4 - 40.5	0.2786
>129	92.52	87.0 - 96.2	36.16	31.2 - 41.3	0.2868
>137	91.84	86.2 - 95.7	38.63	33.6 - 43.8	0.3047
>143	91.16	85.4 - 95.2	38.63	33.6 - 43.8	0.2979
>146	91.16	85.4 - 95.2	39.73	34.7 - 44.9	0.3089
>150	87.07	80.6 - 92.0	45.21	40.0 - 50.5	0.3228
>152	87.07	80.6 - 92.0	47.12	41.9 - 52.4	0.3419
>154	87.07	80.6 - 92.0	47.95	42.7 - 53.2	0.3502
>156	86.39	79.8 - 91.5	50.14	44.9 - 55.4	0.3653

>158	85.03	78.2 - 90.4	51.51	46.2 - 56.7	0.3654
>160	83.67	76.7 - 89.3	53.97	48.7 - 59.2	0.3764
>162	83.67	76.7 - 89.3	55.62	50.4 - 60.8	0.3929
>164	80.95	73.7 - 87.0	59.18	53.9 - 64.3	0.4013
>166	80.95	73.7 - 87.0	59.45	54.2 - 64.5	0.404
>173	80.27	72.9 - 86.4	61.64	56.4 - 66.7	0.4191
>175	80.27	72.9 - 86.4	62.47	57.3 - 67.5	0.4274
**>179**	**75.51**	**67.7 - 82.2**	**67.67**	**62.6 - 72.4**	**0.4318**
>181	73.47	65.6 - 80.4	67.95	62.9 - 72.7	0.4142
>185	68.71	60.5 - 76.1	72.05	67.1 - 76.6	0.4076
>187	65.99	57.7 - 73.6	74.79	70.0 - 79.2	0.4078
>191	63.27	54.9 - 71.1	76.71	72.0 - 81.0	0.3998
>202	59.18	50.8 - 67.2	80.27	75.8 - 84.2	0.3945
>208	54.42	46.0 - 62.6	83.01	78.8 - 86.7	0.3743
>210	51.02	42.7 - 59.3	84.93	80.8 - 88.4	0.3595
>214	39.46	31.5 - 47.8	91.78	88.5 - 94.4	0.3124
>225	37.41	29.6 - 45.8	91.78	88.5 - 94.4	0.2919
>231	31.97	24.5 - 40.2	93.70	90.7 - 96.0	0.2567
>237	23.13	16.6 - 30.8	96.99	94.7 - 98.5	0.2012
>243	20.41	14.2 - 27.8	97.26	95.0 - 98.7	0.1767
>254	17.69	11.9 - 24.8	97.26	95.0 - 98.7	0.1495
>260	15.65	10.2 - 22.5	97.53	95.4 - 98.9	0.1318
>266	7.48	3.8 - 13.0	98.90	97.2 - 99.7	0.0638
>289	0.00	0.0 - 2.5	100.00	99.0 - 100.0	0

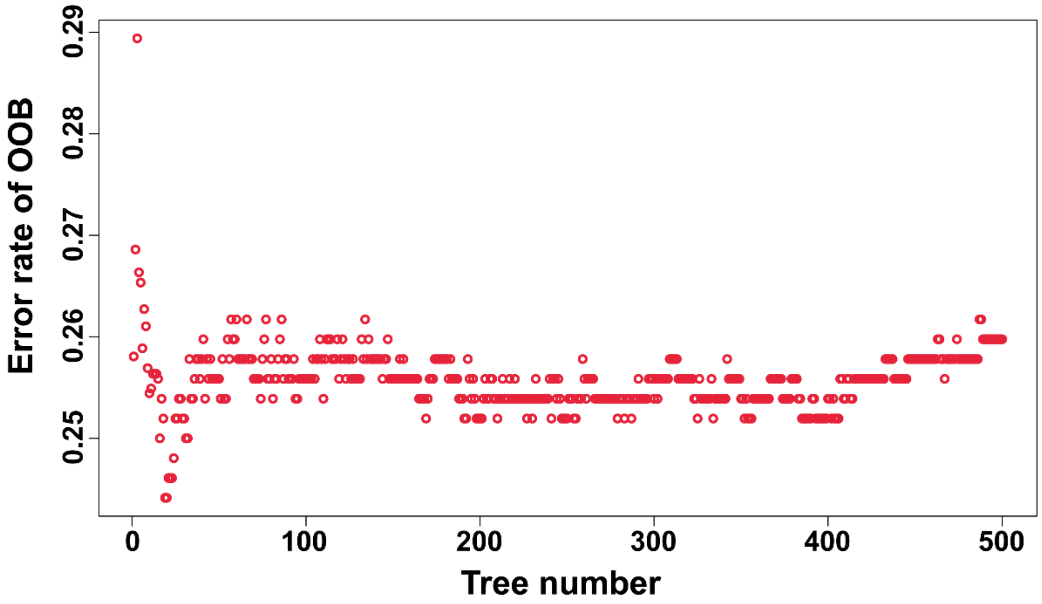

Figure S3. The error rate of OOB according to tree number in a random forest model. Tumor size, tumor location， depth of invasion， tumor differentiation and macroscopic type were incorporated into the random forest model. OOB: out of bag.
